# One‐Pot Base‐Free Suzuki‐Miyaura [^11^C]Methylation: Kinetic and Mechanistic Insights

**DOI:** 10.1002/chem.202503251

**Published:** 2025-11-29

**Authors:** Oscar Moreno, Imad Azzeggarh, Jason P. Holland, Albert D. Windhorst, Jordi Llop

**Affiliations:** ^1^ Department of Radiology and Nuclear Medicine Amsterdam UMC De Boelelaan 1117 Amsterdam Netherlands; ^2^ Radiochemistry and Nuclear Imaging Group CIC biomaGUNE, Basque Research and Technology Alliance (BRTA) San Sebastián Spain; ^3^ Department of Chemistry University of Zurich Zurich Switzerland

**Keywords:** carbon‐11, DFT, kinetic isotope effect, Suzuki‐Miyaura

## Abstract

The Suzuki–Miyaura cross‐coupling reaction is one of the most powerful approaches for forming carbon–carbon bonds in the synthesis of carbon‐11–labeled positron emission tomography (PET) radiotracers. Typically, Suzuki cross‐coupling is performed in the presence of a base to activate the boron reagent and sustain the catalytic cycle. Here, we investigate the capacity of the Suzuki reaction to proceed under one‐pot base‐free conditions for [^11^C]methylation reactions and elucidate the mechanistic features that enable this transformation. Conditions were optimized to successfully achieve one‐pot methylation of a model substrate using [^11^C]CH_3_I in the absence of base in moderate yields. Systematic substrate studies reveal that the reaction is partly governed by the electronic properties of the boron substrate, suggesting an alternative transmetallation pathway in this case. Kinetic studies using [^14^C]CH_3_I, together with kinetic isotope effect (KIE) measurements and density functional theory (DFT) calculations, reveal that oxidative addition remains the rate‐determining step through a heterolytic pathway, consistent with the formation of a cationic Pd(II) intermediate that can undergo transmetallation in the absence of base. Together, these results provide new mechanistic insights into Pd‐mediated [^11^C]C–C bond formation under the unique stoichiometric constraints of radiochemical conditions, expanding the understanding of base‐free cross‐coupling reactions with [^11^C]CH_3_I.

## Graphical abstract

A base‐free Suzuki–Miyaura [^11^C]methylation is achieved in a one‐pot process with [^11^C]CH_3_I on moderate yields. Electronic effects enable transmetallation without boronate activation, while kinetics and DFT show oxidative addition (heterolytic) as the rate‐limiting step.

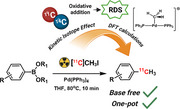

## 1 Introduction

Radiochemistry is the foundation of positron emission tomography (PET), which relies on positron‐emitting radiotracers for imaging [1]. The growing demand for new tracers highlights the need for efficient and versatile radiolabeling methods, with carbon‐11 (^11^C, t_1/2_ = 20.4 min) playing a central role. As carbon is ubiquitous in organic molecules, carbon‐11 enables the labeling of a wide range of compounds [2]. Carbon‐11 radiochemistry relies on fast S_N_2 reactions with [^11^C]methyl iodide ([^11^C]CH_3_I), mostly yielding [^11^C]methoxides, [^11^C]methylamines, and [^11^C]methylthio derivatives [3]. However, these strategies are limited to forming ^11^C–heteroatom bonds and require a heteroatom‐bound methyl group in the final structure, restricting their broader applicability. Alternative strategies allow the formation of C–C bonds, most notably through transition metal–catalyzed cross‐couplings [4, 5]. Such reactions are often described as metal “mediated” rather than truly catalytic since the catalyst is present in excess relative to [^11^C]methyl iodide. Palladium(0)‐mediated reactions using [^11^C]CH_3_I have been widely adapted, including the Stille coupling with organostannanes [6], which tolerates diverse functional groups but often suffers from low molar activity [7]. Negishi couplings with organozinc intermediates expand the scope but require in situ zincate preparation, limiting practicality [8]. A general [^11^C]methylating reagent has since been developed to bypass this need [9]. In contrast, Suzuki‐Miyaura cross‐coupling with boron substrates offers greater stability, lower toxicity, and broader accessibility [4, 10, 11]. Hostetler et al. demonstrated [^11^C]methylation of aryl boronic acids and esters *via* Pd(dppf)Cl_2_‐mediated Suzuki coupling [12]. However, yields were inconsistent in a one‐pot setup due to competing base reactions with [^11^C]CH_3_I. To overcome this limitation, the reaction can be split into two sequential steps: oxidative addition of [^11^C]methyl iodide to the Pd complex, followed by introduction of the aryl boron substrate and base. In this scenario, we hypothesized that, under radiochemical conditions, Suzuki coupling may proceed stoichiometrically without a base that turns over the catalytic cycle, allowing for one‐pot chemistry. Indeed, previous reports suggest that base‐free conditions can improve radiochemical yields (RCYs) [13]. Guided by this, here we explored the feasibility of one‐pot base‐free [^11^C]methylation in different boron substrates *via* Suzuki coupling with [^11^C]methyl iodide. Additionally, we used isotopic labelling with [^14^C]CH_3_I and applied Density Functional Theory (DFT) calculations to understand the reaction kinetics and mechanism of this alternative approach.

## 2 Results and Discussion

**SCHEME 1:**
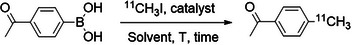
Model reaction.

To establish the standard conditions for a base‐free Suzuki coupling, we selected the radiolabelling of 1‐(4‐([^11^C]methyl)phenyl)ethan‐1‐one from (4‐acetylphenyl)boronic acid and [^11^C]methyl iodide as a model reaction (Scheme 1) (Table 1). We first evaluated the effect of different palladium catalysts on the radiochemical yield, determined by radio‐high performance liquid chromatography (radio‐HPLC) analysis of the crude product. The Pd_2_(dba)_3_ + P(o‐tolyl)_3_ system, previously identified by Doi and coworkers as highly effective for Suzuki coupling with [^11^C]CH_3_I, gave negligible yields under base‐free conditions (entries 1–3) [14]. Similarly, Pd(PPh_3_)_2_Cl_2_ (entry 4) and Pd(dppf)_2_Cl_2_ (entry 5) did not produce the desired product in our hands. The first evidence of conversion was obtained when using Pd(PPh_3_)_4_ at 40°C for 20 min, giving 11% yield (entry 6). Raising the temperature to 80°C increased the yield to 53% in just 10 min (entry 7), while a further increase to 120°C in DMF slightly reduced the yield to 46% (entry 8). Lowering the boronic acid amount from 100 µmol to 10 µmol gave a comparable yield of 50 ± 15% (entry 9, n = 3), whereas shortening the reaction time to 5 min significantly decreased conversion to 13% (entry 10). From these results, the standard conditions for the base‐free Suzuki coupling were defined as follows: Pd(PPh_3_)_4_ (2.5 µmol) and 10 µmol boron substrate in THF at 80°C for 10 min. Clearly, these conditions result in a lower radiochemical yield compared to [^11^C]methylation of aryl boronic acids with base [12]. The addition of Na_2_CO_3_ supported this observation, significantly increasing the yield to 93%, as expected (entry 11). Interestingly, however, the absence of base does not completely suppress the reaction, allowing for radiolabeling under base‐free conditions. Although water can sometimes accelerate Suzuki‐Miyaura‐type cross‐coupling reactions, in our study we opted to maintain anhydrous conditions to simplify the interpretation of further kinetic and mechanistic data under radiochemical conditions.

**TABLE 1:** Screening of reaction conditions for the one‐pot base‐free Suzuki cross‐coupling with [^11^C]methyl iodide based on Scheme 1.

Entry	Boron substrate (µmol)	Catalyst	Solvent	T (°C)	Time (min)	RCY^[^ ^a^ ^]^	N
1	100	Pd_2_(dba)_3_	THF	80	10	0%	1
2	100	Pd_2_(dba)_3_	DMF	150	10	0%	1
3	100	Pd_2_(dba)_3_ + P(*o*‐tolyl)_3_	THF	80	10	3%	1
4	100	Pd(PPh_3_)_2_Cl_2_	THF	80	10	0%	1
5	100	Pd(dppf)Cl_2_	THF	80	10	0%	1
6	100	Pd(PPh_3_)_4_	THF	40	20	11%	1
7	100	Pd(PPh_3_)_4_	THF	80	10	53%	1
8	100	Pd(PPh_3_)_4_	DMF	120	10	46%	1
9	10	Pd(PPh_3_)_4_	THF	80	10	50±15%	3
10	10	Pd(PPh_3_)_4_	THF	80	5	13%	1
11	10	Pd(PPh_3_)_4_	THF	80	10	93%^[^ ^b^ ^]^	1
12	10	Pd(PPh_3_)_4_	THF	80	10	31±5%^[^ ^c^ ^]^	2
13	10	Pd(PPh_3_)_4_	THF	80	10	23±1%^[^ ^d^ ^]^	2

^[a]^ Radiochemical yield based on HPLC analysis of the crude product, average ± standard deviation;

^[b]^ Na_2_CO_3_ added (20 µmol);

^[c]^ nonradioactive CH_3_I (2.5 µmol) added;

^[d]^ nonradioactive CH_3_I (10 µmol) added.

To examine whether an excess of catalyst facilitates base‐free Suzuki reactions under radiochemical conditions, we compared stoichiometric and catalytic regimes using nonradioactive methyl iodide as a carrier. Under stoichiometric conditions (1:1 methyl iodide:Pd), the conversion decreased to 31 ± 5% (entry 12) and dropped further to 23 ± 1% when methyl iodide and boron substrate were used in equal amounts (catalytic conditions; entry 13). Together, these findings indicate that although the absence of base slows down the overall kinetics as expected—likely by reducing activation of the boron species—the unique conditions found in radiochemical reactions (i.e., excess of catalyst compared to methyl iodide) favor base‐free Pd‐mediated Suzuki cross‐coupling.

Building on these results, we next investigated the reactivity of various boron substrates in the absence of base. Under standard Suzuki coupling conditions, the base is thought to facilitate the transmetallation step by activating the boron species into a more reactive boronate anion, which can interact more readily with the Pd(0) centre [15]. As a result, the overall reactivity of the boron substrate is typically less dependent on its substituents than that of the aryl halide. In contrast, under base‐free conditions, we hypothesized that a less‐activated boron species can be more sensitive to electronic effects, with reactivity partially governed by the nature of the substituents. To test this, we screened a series of simple aryl boronic acids and pinacol esters bearing different functional groups under the standard base‐free conditions to obtain the corresponding [*methyl*‐^11^C]aryls products (Table 2). In general, boronic acids containing electron‐donating groups (EDGs) gave only moderate to low radiochemical yields (entries 1–2), whereas pinacol esters consistently resulted in slightly higher yields despite the greater stability of the ester group (entries 3–5). Low yields for OH‐ and NH_2_‐bearing substrates (entries 3, 4) likely reflect their strong electron‐donating nature and Pd‐coordination, both of which can hinder base‐free transmetallation. Substrates with electron‐withdrawing groups (EWGs) afforded moderate to good yields (entries 6–15), particularly with strong EWGs (entries 6–8) and sterically hindered substrates (entry 9). No positional effect was observed for nitro groups placed in either *para‐* or *meta‐*positions (entries 7–8). An iodo‐substituted substrate failed to yield the product, likely due to self‐coupling at the iodo position (entry 10). Notably, substrates bearing a pyrazole ring (entries 14–15) gave higher yields than the benzene analogue (entry 13). In this case, the N‐H pyrazole substrate most likely coordinates to Pd(0) *via* the tautomer, giving lower conversion than the methyl analogue. These results demonstrate that even without formation of a boronate anion, neutral boronic acids and esters partially undergo transmetalation with Pd, directly transferring the aryl group in the absence of base.

**TABLE 2:** Radiochemical yields (average values ± SD) obtained for different boron substrates.

	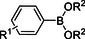		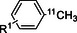
Entry	R^1^	R^2^	RCY (%)^[^ ^a^ ^]^
1	4‐Methoxy	H	12 ± 5%^[^ ^b^ ^]^
2	4‐Methyl	H	9 ± 2%^[^ ^b^ ^]^
3	4‐Hydroxy	pinacol	11 ± 1%^[^ ^c^ ^]^
4	4‐Amino	pinacol	25 ± 8%^[^ ^b^ ^]^
5	4‐dimethylamino	pinacol	14 ± 6%^[^ ^c^ ^]^
6	4‐Acetyl	H	50 ± 15%^[^ ^c^ ^]^
7	4‐Nitro	H	35 ± 9%^[^ ^c^ ^]^
8	3‐Nitro	H	35 ± 5%^[^ ^c^ ^]^
9	2‐Bromo	H	25 ± 7%^[^ ^c^ ^]^
10	4‐Iodo	H	0%^[^ ^b^ ^]^
11	4‐Formyl	H	9 ± 3%^[^ ^b^ ^]^
12	4‐Carboxyl	pinacol	4 ± 2%^[^ ^b^ ^]^
13	4‐Methyl ester	pinacol	18 ± 8%^[^ ^b^ ^]^
	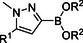		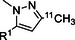
14	4‐Methyl ester	pinacol	57 ± 16%^[^ ^c^ ^]^
	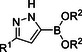		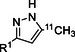
15	4‐Ethyl ester	pinacol	25 ± 6%^[^ ^c^ ^]^

Reagents and conditions: [^11^C]CH_3_I, Pd(PPh_3_)_4_, THF, 80°C, 10 min.

^[a]^ Radiochemical yield based on HPLC analysis of the crude product, average ± standard deviation.

^[b]^ n = 2.

^[c]^ n = 3.

To study the electronic effects further, we plotted the radiochemical yield of each substrate against the reported Hammett constant (σ) of its substituent (Figure S17)[16]. A weak positive correlation emerged for substrates with electron‐withdrawing groups, while electron‐donating groups correlated with reduced reactivity. Although modest, the trend suggests that under base‐free conditions the boron substrate may undergo transmetallation *via* an alternative pathway (i.e., electrophilic substitution transmetallation)[17], in which EWGs enhance the Lewis acidity of the boron center and facilitate aryl transfer to Pd. Conversely, electron‐rich boron substrates may transfer the aryl group less efficiently under this mechanism, thereby appearing less reactive.

**SCHEME 2:**
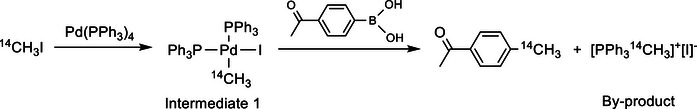
Proposed reaction scheme for the kinetic studies using [^14^C]CH_3_I showing formation of intermediates and by‐products.

**FIGURE 1:**
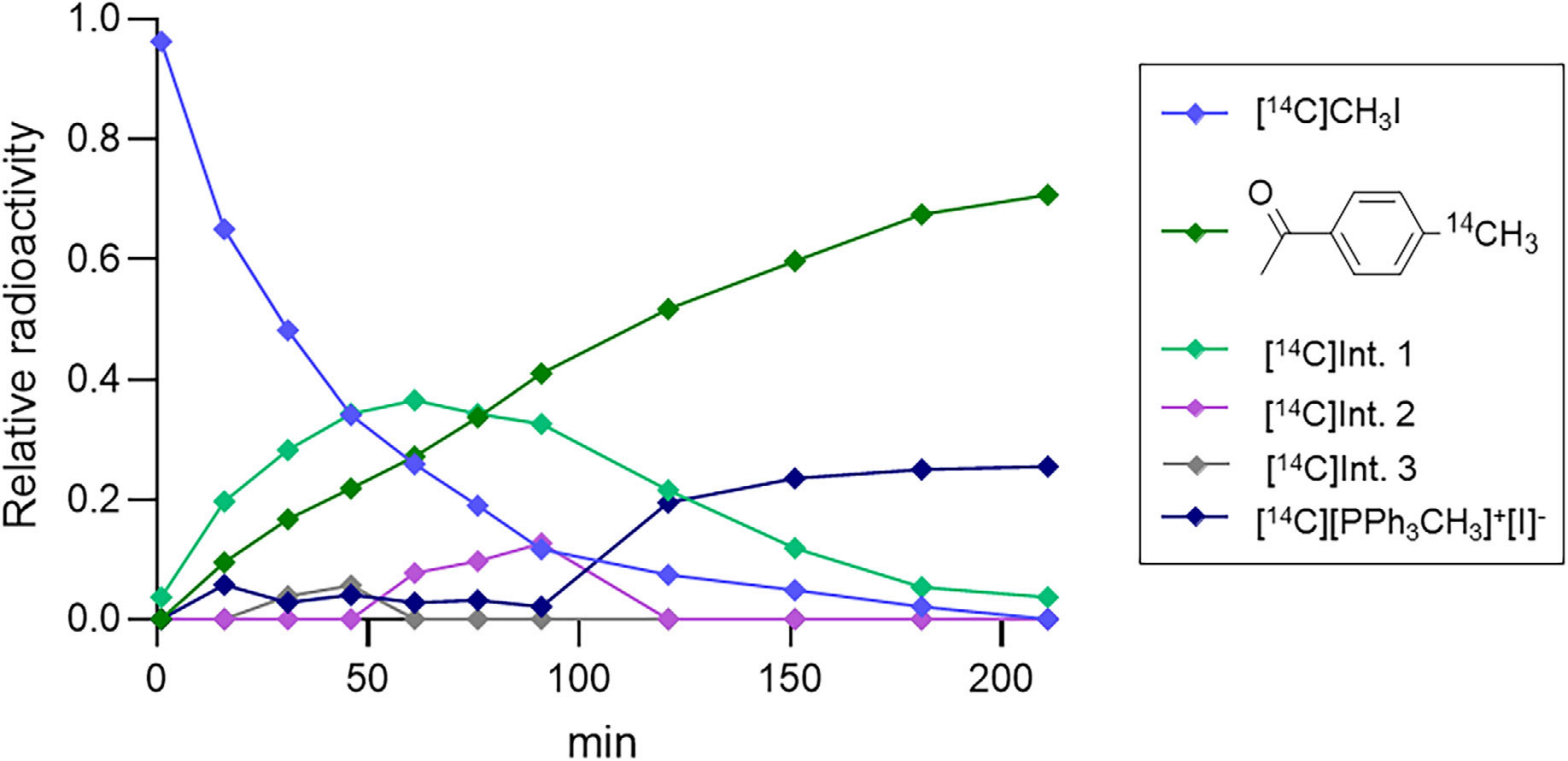
Reaction profile of the base‐free Pd(PPh_3_)_4_‐mediated Suzuki coupling followed by radio‐HPLC. Time course of [^14^C]CH_3_I consumption and appearance of intermediates and products under base‐free conditions at 40°C.

Taking advantage of the high sensitivity of radiochemical detection as well as isotopic mass differences, we anticipated the possibility of identifying reaction products and by‐products with exceptional precision, thereby enabling kinetic and mechanistic investigations. First, to understand how the absence of base affects the reaction kinetics, we performed experiments with long‐lived carbon‐14 methyl iodide ([^14^C]CH_3_I) and (4‐acetylphenyl)boronic acid as a model substrate (Scheme 2). The evolution of the main radioactive species in the crude reaction mixture was monitored over time by radio‐HPLC (Figure 1). Under base‐free conditions at 40°C, [^14^C]CH_3_I consumption followed pseudo‐first‐order kinetics (*k* = 0.02 s^−1^, R^2^ = 0.99, n = 1), with complete conversion observed only after ∼200 min. Three radioactive intermediates were detected: one major (Intermediate 1) and two minor species (Intermediates 2 and 3), which remained unidentified.

**FIGURE 2:**
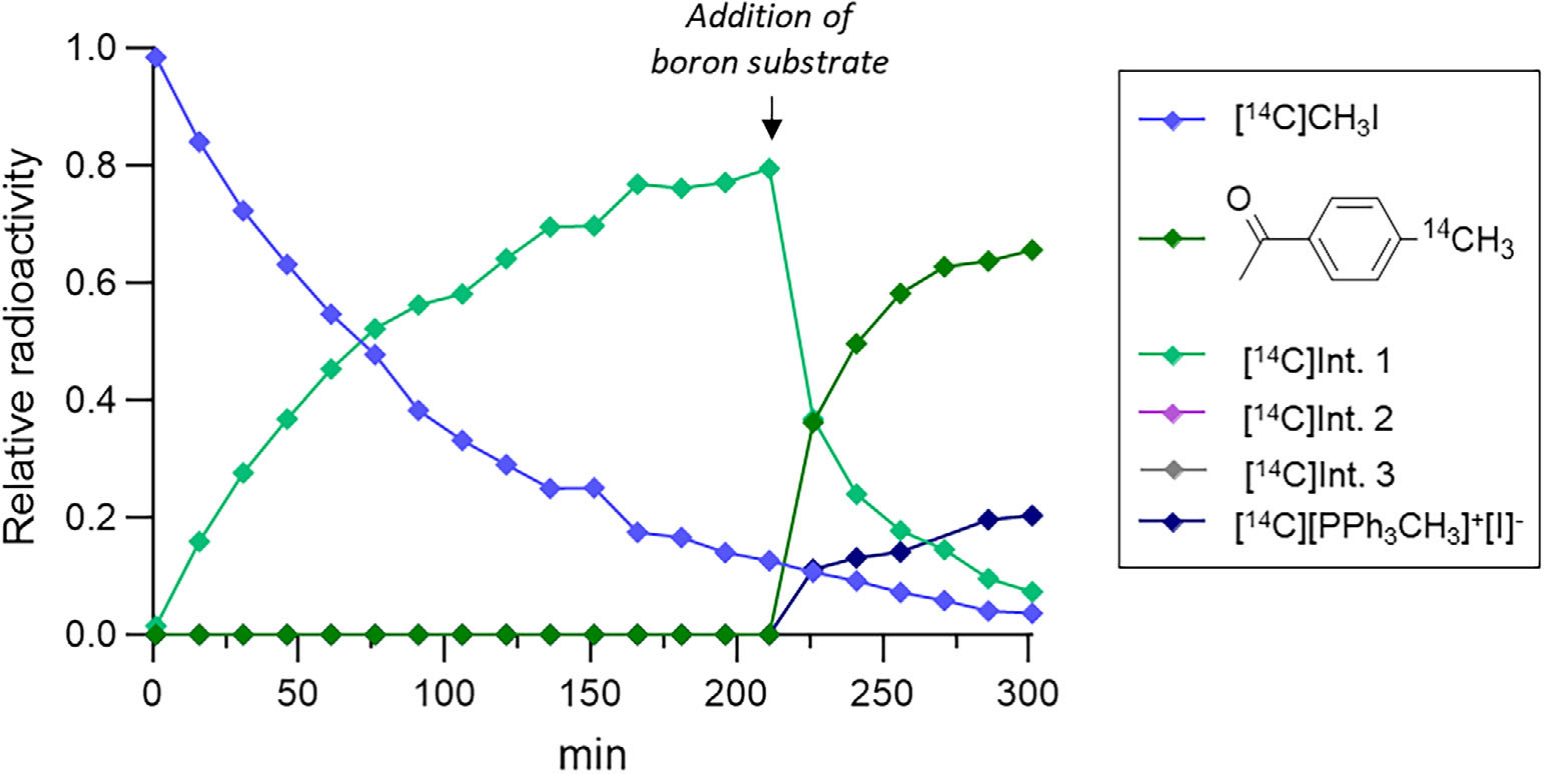
Reaction profile of the base‐free Pd(PPh_3_)_4_‐mediated Suzuki coupling followed by radio‐HPLC. Time‐course with delayed addition of (4‐acetylphenyl)boronic acid at 211 min, showing rapid consumption of Intermediate 1 and formation of the [*methyl*‐^14^C]aryl product together with the phosphonium salt by‐product.

Next, to isolate the oxidative addition step, the reaction was studied in the absence of boron substrate (Figure 2). In this case, Intermediate 1 was identified as the oxidative addition complex Pd(PPh_3_)_2_CH_3_I, and this conclusion was confirmed by coinjection with the corresponding nonradioactive standard. When boronic acid was subsequently introduced (after 211 min), Intermediate 1 was immediately converted into the [*methyl*‐^11^C]aryl product along with the formation of a by‐product identified as the phosphonium salt [PPh_3_CH_3_]^+^[I]^−^, whose identity was confirmed by comparison with the nonradioactive reference compound. Notably, Intermediate 2 was not observed prior to boron substrate addition, suggesting it represents a transient transmetallation complex rapidly consumed during product formation. Altogether, these results indicate that the oxidative addition remains the rate‐determining step (RDS) under base‐free conditions, as in standard Suzuki coupling [18]. Note that in the absence of boron substrate, full conversion of [^14^C]CH_3_I into Intermediate 1 required >200 min, whereas ∼50% of Intermediate 1 was consumed immediately upon addition of boron substrate, consistent with a much faster transmetallation step.

**SCHEME 3:**
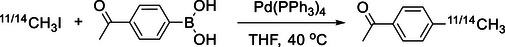
Reaction scheme for the kinetic isotope effect (KIE) study of the base‐free Pd(PPh_3_)_4_‐mediated Suzuki coupling using [^11^C/^14^C]CH_3_I and (4‐acetylphenyl)boronic acid in THF at 40°C.

**FIGURE 3:**
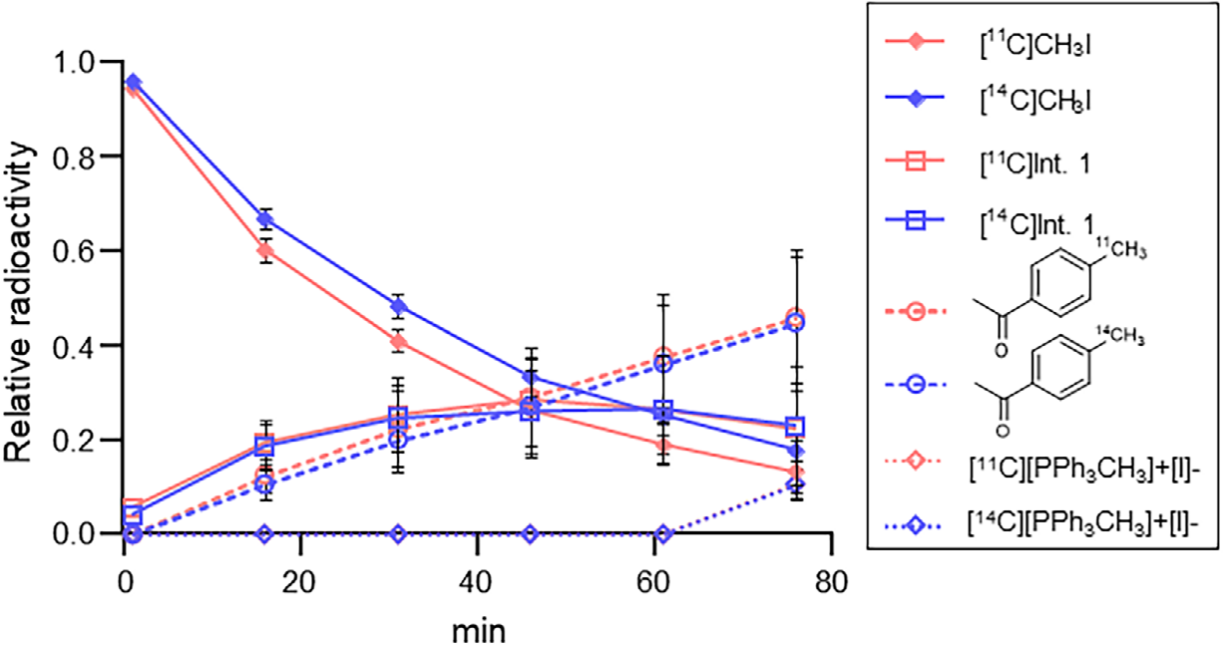
Kinetic isotope effect (KIE) study. Reaction progress was monitored by radio‐HPLC, showing time‐dependent consumption of [^11^C/^14^C]CH_3_I and formation of the corresponding [*methyl*‐^11/14^C]aryl product and intermediates.

This conclusion was reinforced by an isotopic study, where the simultaneous monitoring of [^11^C]CH_3_I and [^14^C]CH_3_I within the same reaction vessel revealed a measurable difference in reaction rates (i.e., kinetic isotope effect) (Scheme 3) (Figure 3). As shown in the relative radioactivity graph, [^11^C]CH_3_I is consumed more rapidly than [^14^C]CH_3_I, leading to a corresponding faster formation of the [*methyl*‐^11^C]aryl product. The observed isotope effect (KIE ^11^C/^14^C = 1.23 ± 0.04, n = 3) provides direct experimental evidence that the cleavage of the C–I bond during oxidative addition still governs the overall reaction rate in base‐free conditions.

**FIGURE 4:**
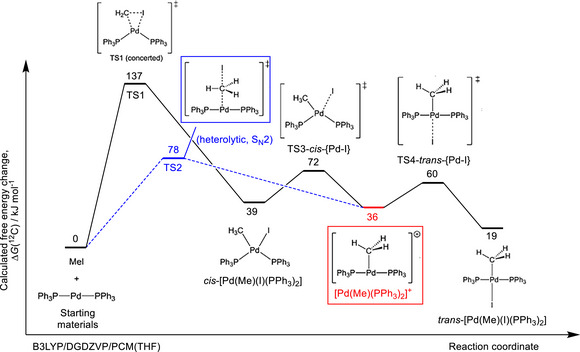
DFT‐calculated free energy profile (B3LYP/DGDZVP, THF continuum solvent) for the oxidative addition of CH_3_I to Pd(PPh_3_)_2_. Two pathways were considered: a concerted transition state (TS1, 137 kJ mol^−1^) and a heterolytic S_N_2‐type transition state (TS2, 78 kJ mol^−1^). The heterolytic pathway is energetically favored and leads directly to the tri‐coordinated cationic intermediate [Pd(Me)(PPh_3_)_2_]⁺ (red box), in agreement with experimental observations. Subsequent equilibria with *cis*‐ and *trans*‐[Pd(Me)(I)(PPh_3_)_2_] isomers are predicted to occur via low‐energy barriers.

To better understand our experimental results, we employed DFT calculations (B3LYP/DGDZVP with THF as a continuum solvent) to examine the oxidative addition of CH_3_I to the Pd(0)‐complex Pd(PPh_3_)_2_ *via* either a concerted or heterolytic pathway (Figure 4). Interestingly, the obtained calculations revealed a barrier of ∼78 kJ mol^−1^ proceeding through a heterolytic pathway, likely the rate‐determining step of the reaction, resembling the mechanistic features described in earlier methyl‐palladium studies [19]. Conversely, the concerted transition state lies 137 kJ mol^−1^ above the ground‐state reactants and 59 kJ mol^−1^ higher than the heterolytic transition state. Importantly, the heterolytic pathway directly furnishes the Pd(II) tricoordinated cationic intermediate [Pd(Me)(PPh_3_)_2_]^+^, which is well positioned for subsequent reaction with the arylboronic acid in the transmetallation step. In the absence of a suitable boron partner, the iodide ligand may remain bound to Pd(II), giving rise to *cis*‐ and *trans*‐[Pd(Me)(I)(PPh_3_)_2_] isomers. Calculations suggest these species are in equilibrium with [Pd(Me)(PPh_3_)_2_]^+^ *via* low‐energy barriers (33.6 and 41.6 kJ mol^−1^, respectively), with the Pd–I bond predicted to be the most labile. To compare with experimental isotope studies, we also calculated the reaction coordinate for oxidative addition using ^11^C‐ and ^14^C‐CH_3_I. However, no meaningful differences were observed, likely because the experimental isotope effect is modest and the associated energetic differences between transition states fall within the intrinsic accuracy of DFT (typically ∼5–10 kJ mol^−1^) [20]. While higher‐level methods may eventually resolve these small isotope effects in Pd–C bond formation, such calculations are currently impractical for transition states of this size and complexity.

## 3 Conclusion

In this explorative study, we demonstrated that Suzuki–Miyaura [^11^C]methylation can proceed under one‐pot, base‐free conditions owing to the unique stoichiometry of radiochemical systems. Kinetic and mechanistic analyses reveal that, although the absence of base slows the overall reaction, C–C bond formation remains feasible and is partly governed by the electronic properties of the boron substrate. The data support an alternative, electronically driven transmetallation pathway operating without boronate activation. Combined kinetic isotope effect studies and DFT calculations confirm that oxidative addition remains the rate‐determining step, proceeding *via* a heterolytic pathway to a cationic Pd(II) intermediate. These findings highlight the valuable role of isotopic labeling in kinetic and mechanistic studies, offering new insights into Pd‐mediated cross‐coupling beyond conventional base‐assisted catalysis.

## Conflicts of Interest

The authors declare no conflict of interest.

## Supporting information




**Supporting File 1**: Experimental details can be found in the supporting information. The authors have cited additional references within the Supporting Information[20–23].

## Data Availability

The data that support the findings of this study are available from the corresponding author upon reasonable request.
