# Supplementary material for: One‐Pot Base‐Free Suzuki‐Miyaura [11C]Methylation: Kinetic and Mechanistic Insights
Source: Chemistry. 2025 Nov 29;32(1):e03251. doi: 10.1002/chem.202503251 (PMC12759168; doi:10.1002/chem.202503251)
Supplement: Supplementary file 1 — Supporting File 1: Experimental details can be found in the supporting information. The authors have cited additional references within the Supporting Information[20–23]. [file CHEM-32-e03251-s001.docx]

Supporting information

**One-pot base-free Suzuki-Miyaura [¹¹C]methylation: kinetic and mechanistic insights**

Oscar Moreno,^*,a,c^ Imad Azzeggarh,^a^ Jason P. Holland,^b^ Albert D. Windhorst^a^ and Jordi Llop^*,c^.

Amsterdam UMC, Vrije Universiteit, Department of Radiology & Nuclear Medicine, De Boelelaan 1117, Amsterdam, The Netherlands

Department of Chemistry, University of Zurich, Winterthurerstrasse 190, Zurich CH-8057, Switzerland

CIC biomaGUNE, Basque Research and Technology Alliance (BRTA), Paseo Miramón 182, 20014, San Sebastián, Spain

**General considerations**

All commercially available reagents (analytical grade) and solvents (analytical grade) were obtained from Sigma Aldrich (Saint Louis, USA) or Fluorochem (Glossop, UK), unless stated otherwise, and used without further purification. *Tetrakis*(triphenylphosphine)palladium(0) was purchased from Strem (Newburyport, USA). [^14^C]CH_3_I was purchased from ViTrax Radiochemicals (Placentia, USA) as a toluene solution over copper (59.2 MBq/mL) and used without further purification.

For the one-pot base-free Suzuki coupling, HPLC analysis was performed by using a Shimadzu system consisting of a LC-20AT liquid chromatography module, SIL-20A HT auto sampler, SPD-20A UV/VIS detector, CTO-20A column oven, DGU-20A5R degassing unit, CBM-20A communication bus module, a Elysia Raytest GABI Nova radiodetector, and using LabSolutions 5.85 software. An Alltima C18 column (4.6×250 mm, 5 µm) (VWR International, Leuven, Belgium) was used as the stationary phase and a ultrapure water/acetonitrile gradient mixture (0 min: 40% ACN; 0-7,5 min: 64% ACN; 7,5-8 min: 90% ACN; 8-14 min: 90% ACN; 14-14.01 min: 40% ACN; 14,01-20 min: 40% ACN) in 0.1% trifluoroacetic acid as the mobile phase at a flow rate of 1 mL/min.

For the kinetic experiments with [^14^C]CH_3_I, HPLC analysis was performed by using an Agilent 1200 Series HPLC system equipped with a radiometric detector (Gabi, Raytest), a UV detector (wavelength = 254 nm) and a β-radioactivity flow detector (Ramona Star, Raytest) with an internal solid scintillator flow cell, connected in series. A reverse phase silica Teknokroma Tracer Excel 120Å, C8, 0.47×250 mm, 5 µm column (Teknokroma, Spain) was used as the stationary phase and a ultrapure water/acetonitrile gradient mixture (0 min: 40% ACN; 0-7.5 min: 64% ACN; 7.5-8 min: 90% ACN; 8-14 min: 90% ACN; 14-15 min: 40% ACN) in 0.1% trifluoroacetic acid as the mobile phase at a flow rate of 1.5 mL/min.

**Production of [^11^C]CH_3_I**

“Wet chemistry” method

For the one-pot base-free Suzuki coupling, [^11^C]CH_3_I was produced by using the classical “wet chemistry” method at the Tracer Center Amsterdam. In short, [^11^C]CO_2_ (ca. 5 GBq) was produced by a ^14^N(p,α)^11^C nuclear reaction performed in a 0.5% O_2_/N_2_ gas mixture using an IBA Cyclone Kiube cyclotron (Belgium). Subsequently, [^11^C]CO_2_ was transferred to an in-house built synthesis unit and trapped in a LiAlH_4_ solution (0.1 mL, 0.1M in THF) at room temperature. When the transferred activity reached a maximum, the THF was evaporated at 130 °C for 2 minutes under a helium flow of 10 mL/min. Subsequently, an aqueous HI solution (0.2 mL, 57%) was added to the reaction vial and [^11^C]CH_3_I was directly distilled into a vial with a continuous helium flow (20 mL/min), ready to be used in the corresponding radiolabelling.

“Gas-phase” method

For the kinetic isotope effect studies with [^14^C]methyl iodide, [^11^C]CH_3_I was produced by using the “gas-phase” method at the radiochemistry lab of CIC biomaGUNE. In short, [^11^C]CH_4_ was directly generated in an IBA Cyclone 18/9 cyclotron (Belgium) by irradiation of a 5% H_2_/N_2_ gas mixture with 18 MeV protons. The radioactive gas was transferred to a TRACERlab FX_c_ Pro synthesis unit (GE Healthcare), trapped in Carbosphere 60/80 (Alltech Associates, Inc.) at -140 ºC, desorbed by heating at 80 ºC and allowed to react with iodine at 720 ºC to form [^11^C]CH_3_I in a gas circulating process. [^11^C]CH_3_I was selectively retained in a trap containing PorapakTM Q (50-80 mesh, Waters Corporation) at room temperature, while unreacted [^11^C]CH_4_ was recirculated. At the end of the process (8-9 cycles), the PorapakTM Q trap was heated at 190 ºC and [^11^C]CH_3_I was distilled under a continuous helium flow (20 mL/min). The gas stream was passed through a trap containing phosphorous pentoxide and Ascarite II® (20-30 mesh) before being flushed in a 10 mL sealed vial containing 5 mL of anhydrous THF, ready to be used in the corresponding radiolabelling.

**General procedure for the one-pot base-free Suzuki coupling**

A standard experimental setup was employed for the Suzuki coupling, carried out by using a custom-built, in-house synthesis unit (Figure S1). In general, a dried 2 mL reaction vial was charged with the catalyst (2.5 µmoles) and the corresponding boron substrate (10 or 100 µmoles), then sealed and purged with helium (10 mL/min) for 10 minutes prior to starting the reaction. The solvent (500 µL) was then added and [^11^C]CH_3_I (2-3 GBq) was immediately distilled into the solution. Once the radioactivity peaked, the vial was heated at the desired temperature for 5, 10, or 20 minutes, depending on the experimental conditions. After the reaction, the vial was cooled to room temperature, and an aliquot was submitted to HPLC for analysis. The ratio between the area of the peak corresponding to the [^11^C]methylaryl (non-isolated) and the sum of the areas of all the peaks in the radiochromatogram of the reaction crude was used to estimate the radiochemical yield of each compound. Radioactive products were identified by comparison with the corresponding non-radioactive reference compounds. Peaks in the radio chromatogram were automatically corrected for decay during the chromatographic run. Representative chromatograms for the radiolabelling of the different [^11^C]methylaryls are shown (Figures S2-S16).

**Figure S1.** Schematic representation of the setup used for the one-pot base-free Suzuki coupling.

**Chromatograms for the radiolabelling of the boron substrate derivatives**

1-(4-(*methyl*-^11^C)phenyl)ethan-1-one

**Figure S2.** Representative chromatogram of the reaction crude for the radiolabelling of 1-(4-(*methyl*-^11^C)phenyl)ethan-1-one.

1-bromo-2-(*methyl*-^11^C)benzene

**Figure S3.** Representative chromatogram of the reaction crude for the radiolabelling of 1-bromo-2-(*methyl*-^11^C)benzene.

1-(*methyl*-^11^C)-4-nitrobenzene

**Figure S4.** Representative chromatogram of the reaction crude for the radiolabelling of 1-(*methyl*-^11^C)-4-nitrobenzene.

1-(*methyl*-^11^C)-3-nitrobenzene

**Figure S5.** Representative chromatogram of the reaction crude for the radiolabelling of 1-(*methyl*-^11^C)-3-nitrobenzene.

4-(*methyl*-^11^C)benzoic acid

**Figure S6.** Representative chromatogram of the reaction crude for the radiolabelling of 4-(*methyl*-^11^C)benzoic acid.

1-hydroxy-4-(*methyl*-^11^C)benzene

**Figure S7.** Representative chromatogram of the reaction crude for the radiolabelling of 1-hydroxy-4-(*methyl*-^11^C)benzene.

Methyl 4-(*methyl*-^11^C)benzoate

**Figure S8.** Representative chromatogram of the reaction crude for the radiolabelling of methyl 4-(*methyl*-^11^C)benzoate.

1-methoxy-4-(*methyl*-^11^C)benzene

**Figure S9.** Representative chromatogram of the reaction crude for the radiolabelling of 1-methoxy-4-(*methyl*-^11^C)benzene.

1-(*methyl*-^11^C)-4-aminobenzene

**Figure S10.** Representative chromatogram of the reaction crude for the radiolabelling of 1-(*methyl*-^11^C)-4-aminobenzene.

1-iodo-4-(*methyl*-^11^C)benzene

**Figure S11.** Representative chromatogram of the reaction crude for the radiolabelling of 1-iodo-4-(*methyl*-^11^C)benzene.

*N,N*-dimethyl-4-(*methyl*-^11^C)aniline

**Figure S12.** Representative chromatogram of the reaction crude for the radiolabelling of N,N-dimethyl-4-(*methyl*-^11^C)aniline.

1-(*methyl*-^11^C)-4-methylbenzene

**Figure S13.** Representative chromatogram of the reaction crude for the radiolabelling of 1-(*methyl*-^11^C)-4-methylbenzene.

4-(*methyl*-^11^C)benzaldehyde

**Figure S14.** Representative chromatogram of the reaction crude for the radiolabelling of 4-(*methyl*-^11^C)benzaldehyde.

Methyl 1-methyl-3-(*methyl*-^11^C)-1H-pyrazole-5-carboxylate

**Figure S15.** Representative chromatogram of the reaction crude for the radiolabelling of methyl 1-methyl-3-(*methyl*-^11^C)-1H-pyrazole-5-carboxylate.

Ethyl 5-(*methyl*-^11^C)-1H-pyrazole-3-carboxylate

**Figure S16.** Representative chromatogram of the reaction crude for the radiolabelling of Ethyl 5-(*methyl*-^11^C)-1H-pyrazole-3-carboxylate.

**Figure S17.** Simple linear regression between the obtained radiochemical yields and the Hammett constants (σ values) for the different tested substituents. Equation of regression and R^2^ indicated within the graph.

**General procedure for the kinetic studies with [^14^C]CH_3_I and [^11^C]CH_3_I**

To a previously dried 1.5 mL vial containing (4-acetylphenyl)boronic acid (16.4 mg, 100 µmoles) in 100 µL of anhydrous THF was added [^14^C]CH_3_I in toluene (0.6 MBq, 0.27 µmoles) under nitrogen. To this vial, Pd(PPh_3_)_4_ (3 mg, 2.6 µmoles) in 190 µL of anhydrous THF was added and the reaction was started by heating at 40 °C. Aliquots (5 µL) were taken every 15 min and then every 30 min after 91 min from the start of the reaction, and injected into a radio-HPLC for analysis (Figure S18). For the experiment of late addition of the boron substrate, (4-acetylphenyl)boronic acid (16.4 mg, 100 µmoles) in 100 µL of anhydrous THF was added at 211 minutes after starting the reaction and aliquots were taken every 15 min. The relative radioactivity of each [^14^C]radiolabelled species was determined by calculating the ratio of its peak area to the total area of all peaks in the radiochromatogram (Table S1). Peaks with an area below 2% of the total area in each time point were not considered for analysis.

**Figure S18.** Representative crude radiochromatograms in counts per second of the carbon-14 detector at 16 min (top) and 121 min (bottom) after starting the reaction. Retention times: [^14^C]CH_3_I (Rt = 7.0 min), [^14^C]product (Rt = 7.8 min), [^14^C]intermediate 1 (Rt = 11.6 min), [^14^C][PPh_3_CH_3_]^+^[I]^-^ (Rt = 5.6 min), [^14^C]intermediate 3 (Rt = 13.2 min).

| **Rt** | **Compound** | **1 min** | **16 min** | **31 min** | **46 min** | **61 min** | **76 min** | **91 min** | **121 min** | **151 min** | **181 min** | **211 min** |
| --- | --- | --- | --- | --- | --- | --- | --- | --- | --- | --- | --- | --- |
| 3,7 min | Int. 2 |  |  |  |  | 2450.3 | 3440.3 | 6021.8 |  |  |  |  |
| 5,6 min | By-product |  | 320.5 | 471 | 881.8 | 877.8 | 1133.2 | 977.7 | 2107.8 | 2651 | 2481.3 | 2838.3 |
| 4,9 min | Int. 3 |  |  | 658.2 | 1229.9 |  |  |  |  |  |  |  |
| 6,9 min | Methyl iodide | 5500.9 | 3607.4 | 8034.9 | 7366.9 | 8190.6 | 6745.2 | 5542.8 | 796.0 | 558.6 | 209.5 | 194.3 |
| 7,8 min | Product | 45.9 | 528.5 | 2789.4 | 4723.7 | 8601.6 | 11938.1 | 19499.5 | 5598.2 | 6745.3 | 6687.1 | 7892 |
| 11,5 min | Int. 1 | 209 | 1093.5 | 4727 | 7430.4 | 11546.4 | 12153.9 | 15526.6 | 2325.6 | 1349.3 | 535.9 | 416.9 |
| 13,2 min | Not identified |  |  | *244.2* | *375.2* | *610.5* | *771.2* | *523.1* | *93.7* |  |  |  |
| **TOTAL area** |  | 5755.8 | 5549.9 | 16924.7 | 22007.9 | 32277.2 | 36181.9 | 48091.5 | 10921.3 | 11304.2 | 9913.8 | 11341.5 |

**Table S1.** Integrated areas for each peak in the radiochromatogram and time point in the experiment with [^14^C]CH_3_I. Peaks below 2% are shown in italics.

To determine the kinetic order and rate of the reaction for [^14^C]CH_3_I, a strong correlation (*R*² = 0.991) was observed when plotting the natural logarithm (ln) of the [^14^C]CH_3_I concentration (ln[[^14^C]CH_3_I]) *versus* time, indicating pseudo-first-order reaction kinetics (Figure S19). The reaction rate was then derived from the slope of the regression line.

**Figure S19.** Regression plot to calculate the rate of reaction for [^14^C]CH_3_I.

For the kinetic isotope effect studies, a similar experimental setup and analysis was employed. In this case, [^11^C]CH_3_I in THF (0.2-0.4 MBq) was also added to the reaction mixture and experiments were run in triplicate. To calculate the relative radioactivity of [^11^C]radiolabelled species, the relative area was decay-corrected to the retention time of each peak. To determine the carbon-11/14 kinetic isotope effect (KIE(^11/14^C)) for the methyl iodide (^11/14^C-KIE_MeI_) we first calculated the rate of [^14^C]CH_3_I and [^11^C]CH_3_I in each reaction, as described before, and then determined the KIE as follows:

$$\mathrm{KIE}(^{11}C/^{14}C) = \frac{k[¹¹C]CH₃I}{k[¹⁴C]CH₃I}$$

**Density functional theory (DFT) calculations**

All calculations were conducted using density functional theory (DFT) as implemented in the Gaussian16 Revision A.03 suite of *ab initio* quantum chemistry programs (Table S2).^[1]^ Normal self-consistent field (SCF) and geometry convergence criteria were employed throughout. All structures were optimised in solution phase using a polarisable continuum model (PCM) without symmetry constraints. Calculations were performed by using the B3LYP exchange-correlation functional^[2],^^[3]^ and the DGDZVP basis set.^[4]^ Solvated phase calculations were implemented by using the SCRF keyword with default parameters and selecting THF as the solvent (dielectric constant, ε = 7.34257). The choice of solvation model reflects the conditions employed in the radiosynthesis. Harmonic frequency analysis based on analytical second derivatives was used to characterize the optimized structures as local minima or first order saddle points (transition states) on the potential energy surface.

**Table S2.** Calculated energetics associated with the oxidative addition of MeI to the Pd(PPh_3_)_2_ reagent *via* either a concerted or heterolytic pathway.

| **Compound** | **Δ*G* / kJ mol^-1^** | **Δ*H* / kJ mol^-1^** | **–*T*Δ*S* / kJ mol^-1^** | ***i* (cm^-1^)** |
| --- | --- | --- | --- | --- |
| [Pd(Me)(I)(PPh_3_)_2_]-TS1 (concerted) | 136.5  136.4  136.7 | 81.9  81.9  82.0 | 54.6  54.5  54.6 | *i*296.5  *i*304.4  *i*282.8 |
| *cis*-[Pd(Me)(I)(PPh_3_)_2_] | 38.7  38.7  38.7 | -29.2  -29.2  -29.2 | 67.9  67.9  67.9 | N/A |
| *trans*-[Pd(Me)(I)(PPh_3_)_2_] | 18.5  19.9  19.6 | -45.7  -45.6  -45.7 | 64.2  65.6  65.3 | N/A |
| [Pd(Me)(I)(PPh_3_)_2_]-TS2 (heterolytic, S_N_2) | 78.0  77.8  78.2 | 30.3  30.2  30.4 | 47.7  47.6  47.8 | *i*348.4  *i*363.1  *i*323.8 |
| *cis*-[Pd(Me)(I)(PPh_3_)_2_]-TS3 | 72.3 | 6.2 | 66.1 | *i*19.9 |
| *trans*-[Pd(Me)(I)(PPh_3_)_2_]-TS4 | 60.1 | -17.4 | 77.5 | *i*43.1 |
| [Pd(Me)(PPh_3_)_2_]^+^ | 35.8  30.9  43.4 | 4.4  6.1  3.1 | 31.4  24.8  40.3 | N/A |

Note: Energetic data present the relative change with respect to the starting materials of the free energy, Δ*G* / kJ mol^-1^, enthalpy, Δ*H* / kJ mol^-1^, and –*T*Δ*S* / kJ mol^-1^ (at *T* = 298.15 K) from DFT calculations using the B3LYP/DGDZVP methodology combined with a continuous solvation model of the solvent THF. Separate data are presented for calculations involving isotopic substitution of the methyl carbon atom for ^12^C (black), ^11^C (red), and ^14^C (blue). Imaginary frequencies associated with the optimised transition states are also presented, *i* (cm^-1^). N/A = not applicable.

The nature of transition states (first order saddle points characterized with one single imaginary vibrational frequency) was confirmed to connect the appropriate minima by animation of the vibrational mode combined with re-optimisation after minor molecular displacement of the mode, as well as by using intrinsic reaction coordinate (IRC) calculations. Potential energy scans with full geometry relaxation were used to identify the transition states (TS3 and TS4; see mechanistic scheme) and dissociation energies for dissociation of the Pd-I bond in the *cis*-[Pd(Me)(I)(PPh_3_)_2_] or *trans*-[Pd(Me)(I)(PPh_3_)_2_] intermediates. Optimised structures were analysed by using Chemcraft (version 1.8, build 536b).

1. Frisch, M. J. et al. Gaussian16 (Revision A.03), Gaussian Inc. Wallingford CT. Gaussian16 (Revision A.03) (2016).
2. Becke, A. D. (1993). *The Journal of Chemical Physics*, 98(7), 5648–5652.
3. Stephens, P. J., Devlin, F. J., Chabalowski, C. F., & Frisch, M. J. (1994). *J. Phys. Chem.* (Vol. 98).
4. Sosa, C., Andzelm, J., Elkin, B. C., Wimmer, E., Dobbs, K. D., & Dixon, D. A. (1992). *J. Phys. Chem* (Vol. 96).
